# Encouraging children to eat more fruit and vegetables: Health vs. descriptive social norm-based messages

**DOI:** 10.1016/j.appet.2016.01.031

**Published:** 2016-05-01

**Authors:** Maxine Sharps, Eric Robinson

**Affiliations:** Institute of Psychology, Health and Society, Department of Psychological Sciences, University of Liverpool, Liverpool, UK

**Keywords:** Social eating, Social norm messages, Social norms, Fruit and vegetable intake, Perceived eating norms

## Abstract

Traditional intervention approaches to promote fruit and vegetable consumption outline the health benefits of eating fruit and vegetables. More recently, social norm-based messages describing the healthy eating habits of others have been shown to increase fruit and vegetable intake in adults. Here we report two experimental studies which investigated whether exposure to descriptive social norm-based messages about the behaviour of other children and health-based messages increased fruit and vegetable intake in young children. In both studies children were exposed to messages whilst playing a board-game. After exposure to the messages, children were able to consume fruit and vegetables, as well as high calorie snack foods. Although findings were inconsistent across the two individual studies, in a pooled analysis we found evidence that both health messages and descriptive social norm-based messages increased children's fruit and vegetable intake, relative to control condition messages (*p* < .05). Whether descriptive social norm-based messages can be used to promote meaningful changes to children's dietary behaviour warrants further study.

## Highlights

• Descriptive social norm-based and health messages were examined.
• Health messages increased fruit and vegetable intake.
• Descriptive social norm-based messages increased fruit and vegetable intake.

## 1 Introduction

High fruit and vegetable consumption is associated with a reduced risk of major chronic diseases (Bazzano et al., 2002, Hung et al., 2004), however, children eat less fruit and vegetables than recommended (Dennison et al., 1998, Yngve et al., 2005). Eating behaviours are believed to develop through social learning during childhood (Birch and Fisher, 1998, Birch et al., 2007), with the presence of dining companions, such as parents, peers and siblings influencing the development of food preferences and eating behaviours (Birch and Fisher, 1998, Birch et al., 2007, Sharps et al., 2015). Eating behaviours developed during childhood also track into adolescence and adulthood (Kelder et al., 1994, Singer et al., 1995), therefore, understanding how we can encourage children to acquire healthy eating habits is important.

Traditional intervention approaches to encourage fruit and vegetable intake outline the health benefits of eating fruit and vegetables. However, the effectiveness of this approach is unclear. Some studies support that health messages can motivate healthier food choices in adults and children (Bannon and Schwartz, 2006, Lawatsch, 1990, Robinson et al., 2013). For example, in one study, exposure to nutrition messages about apples in a video influenced children to choose an apple rather than a cracker (Bannon & Schwartz, 2006). Likewise, exposing adults to information suggesting that limiting junk food consumption can be beneficial to health, reduced junk food consumption relative to a control condition in a recent study (Robinson, Harris, et al., 2013). However, there are also studies which suggest that, in some contexts, health messages may not be an effective way to increase fruit and vegetable intake (Maimaran and Fishbach, 2014, Musher-Eizenman et al., 2011, Wardle and Huon, 2000). For example, Maimaran and Fishbach (2014) showed that presenting food as instrumental to achieving a goal, for example, outlining the health benefits of eating certain foods, decreased consumption in pre-school children. This may be explained by a form of boomerang effect (Schultz et al., 2007, Werle and Cuny, 2012), whereby increasing the perceived healthfulness of a food reduces consumption. This is also in fitting with suggestions that when a person believes a food is ‘healthy’ it will be less appealing and enjoyable to eat (Raghunathan, Naylor, & Hoyer, 2006).

Although there is mixed evidence regarding the effectiveness of health messages, a significant body of research indicates that eating behaviour can be socially influenced. Adults and children have been shown to adjust their food intake to that of a present peer (Bevelander et al., 2012, Feeney et al., 2011, Robinson and Higgs, 2012). There is also consistent evidence that adults adjust their food intake based on their beliefs about the eating behaviour of others (Pliner and Mann, 2004, Robinson et al., 2014, Robinson et al., 2014, Sharps and Robinson, 2015). The role that beliefs about others' eating behaviour have on eating behaviour has been less thoroughly examined in children. However, in a recent study children were exposed to information suggesting that other children taking part in the study had been eating a large amount of vegetables, and this resulted in children increasing their own vegetable intake (Sharps & Robinson, 2015). Growing evidence suggests that it may be possible to promote healthier eating behaviour in adults through the use of descriptive social norm-based messages. Descriptive social norm-based messages are messages which highlight the healthy eating behaviour of others, and have been shown to influence food intake in adults and adolescents (Robinson et al., 2013, Robinson et al., 2013, Stok et al., 2014). For example, Robinson, Fleming, et al. (2013)found that exposure to a descriptive social norm-based message suggesting that other young adults frequently ate fruit and vegetables, influenced young adults to increase their intake of fruit and vegetables relative to a health message about the benefits of fruit and vegetable consumption. However, there is some evidence that descriptive social norm-based messages may not always be effective in increasing fruit intake (Stok, de Ridder, de Vet, & de Wit, 2012), whilst in other studies, descriptive social norm-based messages have been shown to be no more effective than a health message (Robinson, Harris, et al., 2013) or an instructive message (e.g. have a salad) (Mollen, Rimal, Ruiter, & Kok, 2013). The effect that descriptive social norm-based messages have on the eating behaviour of children has not been investigated. Given that descriptive social normative information (e.g. an information sheet showing the intake of the previous children) has been shown to influence vegetable consumption in young children in a previous study (Sharps & Robinson, 2015), it is plausible that descriptive social norm-based messages about children's fruit and vegetable consumption could be an effective way of encouraging children to ‘fit in’ and eat more fruit and vegetables.

The present studies tested the effect of messages outlining the health benefits of eating fruit and vegetables, and descriptive social norm-based messages suggesting that other children eat fruit and vegetables, on consumption of fruit and vegetables in children aged 6–11years old. We focused on this age range as previous studies have shown that children of this age are socially influenced by their peers when eating (Bevelander et al., 2012, Romero et al., 2009) and conform to descriptive social norms about food intake (Sharps & Robinson, 2015). Across two studies we exposed children to messages about fruit and vegetables as part of an interactive board-game. In line with previous studies in adults, we predicted that children exposed to descriptive social norm-based messages would increase their intake of fruit and vegetables relative to participants in a control condition. Because of inconsistent findings concerning the effect that health messages have on eating behaviour (Bannon and Schwartz, 2006, Lawatsch, 1990, Maimaran and Fishbach, 2014, Robinson et al., 2013), we reasoned that health messages may only have a modest influence on fruit and vegetable consumption.

## 2 Study 1

### 2.1 Method

#### 2.1.1 Participants

143 children (60% females) aged 6–11 years old, (M = 8.75 (SD ± 1.04) were recruited from two Primary schools in North-West England. The sample consisted of 93 healthy-weight and 50 overweight children. Participants were led to believe that the study was looking at how people play board games, and were randomly assigned to one of three conditions; descriptive social norm-based message vs. health message vs. control. In both studies we aimed to recruit at least 40 children per experimental condition. In recent work we have conducted examining social norms and children's vegetable consumption we identified a statistically large effect size (Sharps & Robinson, 2015). Therefore, a sample size of 40 children per condition provided more than adequate statistical power to detect a similar sized effect. The study was approved by the University of Liverpool Research Ethics Committee. Fully-informed parental consent was provided.

#### 2.1.2 Procedure

Study sessions took place during week days between 9am and 3.30pm in UK primary schools. First, the researcher informed the child that the research was about the different ways in which children play games, and the child was seated next to the researcher in front of a board-game. The researcher explained the aim of the game; to move around the board, collect cards and reach the end. Both the child and the researcher had a pack of cards which contained a number and were used to move around the board. Movement around the board was identical in each condition. The child always started first and always won the game. As the child and the researcher moved around the board, they landed on three spaces where they selected a message card. Children's cards contained a message, while the researchers' cards contained an image (fruit and vegetables in the descriptive social norm-based and health message conditions, or animals in the control condition). On selecting a card, the child was required to read the message aloud and explain their interpretation to the researcher. At the end of the game the child was required to explain to the researcher what they had learned during the game, and recited the messages to ensure the researcher knew that the child understood the messages. All children were able to correctly describe the meaning of the messages. The game took approximately 7 min.

Next, the child was informed that there would be a short break before the next game, and the child was presented with the tray of snack foods. The child was informed that they could eat as much of the snack foods as they wished, and was left alone for 7 min. Following the ‘break’, the researcher returned and presented the child with a second game, which involved sorting pictures of fruit and vegetables (e.g. an image of carrots) and high calorie snack foods (e.g. an image of crisps) into one of two piles; fruit and vegetables or sweets and crisps. This enabled the researcher to identify that all children knew what fruit and vegetables were.^1^ Finally, the researcher asked the child what they thought the aims of the study were, completed the questionnaire measures with the child, and measured the child's height and weight.

#### 2.1.3 Messages

Participants were randomly assigned to play one of three board-games. One board contained images of fruit and vegetables, which was used in the descriptive social norm-based message and health message conditions, while the other board contained images of animals, which was used in the control condition. Both boards were identical, except for the images and the name of the game (‘fruit and vegetable towers’ for the descriptive social norm-based message condition and health message condition, and ‘pet shop’ for the control condition). Children were exposed to messages in the form of message cards, which were selected at pre-determined points during the game. In the descriptive social norm-based message condition the messages stated ‘*other children eat lots of fruit and vegetables every day and like them’, ‘other children eat fruit and vegetables every day as snacks’, and ‘other children eat fruit and vegetables at break time’*. In the health message condition the messages stated ‘*fruit and vegetables are really good for you’, ‘fruit and vegetables have lots of vitamins’, and ‘fruit and vegetables make you strong and healthy’*. In the control condition the messages stated *‘all polar bears are left handed’, ‘snails can sleep for up to three years’, and ‘penguins can jump really high in the air’*.

#### 2.1.4 Snack food

Children were provided with four snack foods; one fruit (green seedless grapes, 66 kcal/100 g), one vegetable (carrot sticks, 42 kcal/100 g), and two high calorie snack food items (chocolate chip cookies, 487 kcal/100 g, and ready-salted crisps, 526 kcal/100 g). All foods were purchased from Tesco Ltd (United Kingdom) and were presented in individual bowls. The participants were not made aware that food choice and intake would be examined during the study. The bowls of snack foods were weighed pre and post-consumption to determine the amount of fruit and vegetables, and high calorie snack foods eaten (in grams).

#### 2.1.5 Body weight

Height was measured to the nearest 0.5 cm using a stadiometer (Seca 213, Seca GmbH & Co.) and weight was measured to the nearest 0.1 kg using a digital scale (Seca 813 digital scale, Seca, GmbH & Co.). BMI was calculated as weight (kg)/height (m^2^). Using internationally recognised criteria for children (Cole & Lobstein, 2012) healthy-weight, overweight and obesity were defined based on age and sex-specific BMI cut-off points equivalent to adult BMI of 25–30 kg/m^2^ respectively.

#### 2.1.6 Self-report measures

##### 2.1.6.1 Fruit and vegetable consumption and liking and hunger

The Day in the Life Questionnaire (DILQ) was used to assess usual fruit and vegetable intake in children, as it is a valid and reliable measure for use in children (Edmunds & Ziebland, 2002). The DILQ is a supervised exercise which uses words and pictures to encourage the child to recall and describe a range of activities, including their entire food intake, for the previous day (Edmunds & Ziebland, 2002). We also included measures for the children's liking of fruit and vegetables (e.g. how much do you like fruit/vegetables) with 5 response options ranging from ‘not at all’ to ‘a lot’. These questions were assessed using smiley-face Likert-style scales, and were based on Lally, Bartle, and Wardle (2011).

##### 2.1.6.2 Beliefs about descriptive social norms

To examine whether the messages children were exposed to influenced their later beliefs about other children's fruit and vegetable intake, they were asked ‘how many fruit and vegetables do you think other children eat every day?’ with responses ‘none’, ‘1’, ‘2–3’, ‘4’, ‘5 or more’.

#### 2.1.7 Main analysis strategy

We planned to conduct one-way ANOVAs to test the main effects of experimental condition (independent variable; descriptive social norm-based messages, health messages, control messages) on the amount of fruit and vegetables, and high calorie snack food eaten in grams (dependent variables). We planned to follow up significant effects of condition with Bonferroni-corrected pairwise comparisons.

### 2.2 Results

No significant differences (*p*s < .05) were found between the conditions for BMI, gender or child age. See Table 1. No children guessed, or came close to guessing the aims of the study.

#### 2.2.1 Food intake

There was a significant main effect of condition on fruit and vegetable intake [F(2,140) = 4.61, *p* = .01, ƞp^2^ = .06]. Children in the health message condition ate significantly more fruit and vegetables than children in the control condition, t(92) = 3.06, *p* = .009, *d* = .63. However, there was no significant difference between the health message condition and the descriptive social norm-based message condition, t(95) = −1.14, *p* = .78, *d* = .23, and no significant difference between the descriptive social norm-based message condition and the control condition, t(93) = 2.00, *p* = .15, *d* = .41. There was no significant main effect of condition on high calorie snack food intake [F(2,140) = .01, *p* = .99, ƞp^2^ < .001]. See Table 2 for mean intake figures for Study 1.

#### 2.2.2 Other variables

In order to examine whether controlling for weight-status, child age, gender, liking of fruit and vegetables, or usual fruit and vegetable intake altered the effect of condition on fruit and vegetable and high calorie snack food intake, we included these variables as covariates in separate ANCOVAs. Controlling for these variables did not alter the effect of condition on the dependent variables. See supplemental material. Furthermore, we also examined whether these variables moderated the effect of condition on fruit and vegetable consumption and high calorie snack food intake. We found no evidence that any of the other variables interacted with message type (*p*s > .05). See supplemental material.

#### 2.2.3 Beliefs about descriptive social norms

To examine whether message type appeared to have a long-term influence on children's beliefs regarding the fruit and vegetable intake of other children (i.e. measured at the end of the study), a one-way ANOVA was conducted. There was no significant main effect of condition on children's beliefs about the amount of fruit and vegetables eaten by other children [F(2,140) = 1.97, *p* = .14, ƞp^2^ = .03]. See Table 1.

### 2.3 Discussion

Exposure to health messages influenced children to increase their fruit and vegetable intake relative to exposure to control messages. However, descriptive social norm-based messages did not significantly increase fruit and vegetable consumption relative to the control condition. There was however a tendency for participants in the descriptive social norm-based message condition to eat more fruit and vegetables than participants in the control condition (see Table 2), so we planned a second study to further examine whether descriptive social norm-based messages can increase fruit and vegetable intake. Although in Study 1 the children in the descriptive social norm-based message condition were exposed to multiple descriptive social norm-based messages and were able to explain what the messages meant to the researcher, our post study questionnaire in Study 1 indicated that these messages did not appear to have a long lasting influence on children's beliefs. In order to address this, in Study 2 we included an additional norm manipulation, whereby, at the end of the board game children in the descriptive social norm-based message condition were shown on a visual scale the amount of fruit and vegetables other children eat (it always indicated that others were eating more than them) as we believed such social comparison may reinforce and motivate adherence to the descriptive social norm-based messages. Another possible explanation for why the descriptive social norm-based messages did not influence fruit and vegetable intake may be due to the norm reference group in the messages i.e. ‘other children’. Research has shown that social ‘distance’ may be an important factor that predicts whether a person conforms to an eating norm (Cruwys et al., 2012). For example, Cruwys et al. (2012) showed that adults only modelled the eating behaviour of salient in group members, i.e. students from their university. Therefore, we changed the norm reference group in Study 2 to a group which was of a closer social distance to the children in the study, i.e. children like you. In addition, in Study 2 we included an extra condition (exposure condition), in order to examine whether merely providing children with information about fruit and vegetables would be sufficient to increase consumption.

## 3 Study 2

### 3.1 Method

#### 3.1.1 Participants

164 children (51% males) aged 6–11 (M = 8.89 SD ± 1.31) were recruited from three primary schools in North-West England. The study consisted of 127 healthy-weight and 37 overweight children. As in Study 1, participants were led to believe that the study was looking at how children play games. The study was approved by the University of Liverpool Research Ethics committee. Fully-informed consent was provided.

#### 3.1.2 Design and procedure

Children were randomly assigned to one of four conditions; descriptive social norm-based message vs. health message vs. exposure vs. control. The same board games were used as Study 1. In addition, at the end of the game we included a visual scale in the descriptive social norm-based message condition only, which was a scale, with anchors ‘none’ and ‘5 or more’ to indicate the amount of fruit and vegetables eaten by other children. The researcher placed a counter described as ‘other children’ under ‘5 or more’ on the scale to show that other children ate a lot of fruit and vegetables, and a counter ‘you’ was placed under ‘1–2 pieces’, to indicate that the child (participant) ate less than other children.^2^ We used the same procedure as in Study 1, except for the inclusion of the additional norm manipulation described above and the inclusion of the exposure condition; within this condition, children played on the ‘fruit and vegetable towers’ board game, and were exposed to facts about fruit and vegetables.

#### 3.1.3 Messages

We altered the messages slightly in the descriptive social norm-based message condition. In the descriptive social norm-based message condition the messages stated; *children like you eat lots of fruit and vegetables every day and like them, children like you eat fruit and vegetables every day as snacks, children like you eat fruit and vegetables at break time.* In the exposure condition the messages stated: *strawberries have seeds on the outside, carrots help you to see in the dark, grapes are actually a berry*. The health messages and control messages remained the same as in Study 1.

### 3.2 Results

No significant differences (*p*s < .05) were found between the conditions for BMI, gender or child age. See Table 3. No children guessed, or came close to guessing the aims of the study.

#### 3.2.1 Food intake

There was no significant main effect of condition on fruit and vegetable intake [F(3,160) = 1.17, *p* = .33, ƞp^2^ = .02] or on high calorie snack food intake [F(3,160) = .54, *p* = .67, ƞp^2^ = .01]. See Table 4 for mean intake figures for Study 2.

#### 3.2.2 Other variables

Controlling for weight-status, child age, gender, liking of fruit and vegetables and usual fruit and vegetable intake as covariates in separate ANCOVAs did not alter the effect of condition for either fruit and vegetable intake, or high calorie snack food intake, see supplemental material. Furthermore, we examined whether weight-status, child age, gender, liking of fruit and vegetables, or usual fruit and vegetable intake moderated the effect of condition on fruit and vegetable intake. There was no significant main effect of weight-status on fruit and vegetable intake [F(1, 156) = 1.22, *p* = .27, ƞp^2^ = .01], or high calorie snack food intake [F(1, 156) = .34, *p* = .56, ƞp^2^ = .002], however there was a significant interaction between condition and weight status on fruit and vegetable intake [F(3,156) = 3.94, *p* = .01, ƞp^2^ = .07]. To follow this interaction up, we next conducted one way ANOVAs in healthy-weight and overweight children separately. There was a significant main effect of condition in healthy-weight children [F(3,123) = 3.42, *p* = .02, ƞp^2^ = .08]. Healthy-weight children in the health message condition tended to eat more fruit and vegetables than healthy-weight children in the control condition, t(65) = 2.70, *p* = .05, *d* = .66. See Supplemental Table 1. However, there were no significant differences between the health message condition and the descriptive social norm-based message condition, t(59) = −2.08, *p* = .24, *d* = .54, or between the health message condition and the exposure condition, t(65) = .41, *p* ≥ .99, *d* = .10. In addition, the descriptive social norm-based message condition did not differ significantly from either the exposure condition, t(58) = −1.74, *p* = .54, *d* = .45, or the control condition, t(58) = .39, *p* ≥ .99, *d* = .10. The exposure condition did not consume significantly more fruit and vegetables than the control condition t(64) = −2.33, *p* = .12, *d* = .57. There was no significant effect of condition on fruit and vegetable intake in overweight children [F(3,33) = 1.46, *p* = .25, ƞp^2^ = .12]. There was no significant interaction between condition and weight status on high calorie snack food intake [F(3,160) = .54, *p* = .66, ƞp^2^ = .01]. We found no evidence that any of the other variables interacted with condition (*p*s > .05). See supplemental material.

#### 3.2.3 Beliefs about descriptive social norms

To examine whether message type influenced children's beliefs regarding the fruit and vegetable intake of other children a one-way ANOVA was conducted. There was a significant main effect of condition [F(3,160) = 6.44, *p* = <.001, ƞp^2^ = .11]. See Table 3. Children in the descriptive social norm-based message condition believed that other children ate more fruit and vegetables than did children in the health message condition, t(80) = 3.37, *p* = .006, *d* = .87, and children in the control condition, t(80) = 4.40, *p* *≤* .001, *d* = .85. However, children in the descriptive social norm-based message condition did not believe that other children ate more fruit and vegetables than children in the exposure condition, t(80) = 1.76, *p* = .48, *d* = .38. In addition, there were no differences between the health message condition and the exposure condition t(80) = −1.88, *p = .36* , *d* = −.42, or the control condition t(80) = .87, *p* ≥ .99, *d* = .20, and no difference between the exposure condition and control condition, t(80) = −2.05, *p* = .24, *d* = −.45.

### 3.3 Discussion

Consistent with Study 1, exposure to descriptive social norm-based messages did not result in a statistically significant increase in children's fruit and vegetable intake relative to a control condition, although there was a tendency for participants to eat slightly more fruit and vegetables in the descriptive social norm-based message condition than in the control condition. However, unlike Study 1, weight status was found to moderate the effect of message type, with healthy-weight children in the health message condition eating more fruit and vegetables than healthy-weight children in the control condition, but with no effect of message type among overweight children. Given that we had only a small number of overweight children in the sample, caution must be taken in interpreting the significant interaction observed in Study 2. Messages which simply provided information (facts) about fruit and vegetables did not significantly increase fruit and vegetable intake relative to the control condition.

## 4 Meta-analysis

In both studies participants in the descriptive social norm-based message conditions did not eat statistically significantly more fruit and vegetables in comparison to the control condition, although this may have been caused by a lack of statistical power. Moreover, we found inconsistent results concerning the effect of health messages and the moderating influence of weight status on messages; in Study 1 weight status did not moderate the effect of a health message on fruit and vegetable consumption, whilst in Study 2 there was evidence of this. To address these inconsistencies we combined data from the health message, descriptive social norm-based message and control message conditions across both studies. We examined the effects of health vs. control messages and descriptive social norm-based vs. control messages in two separate 2 × 2 ANOVAs whilst controlling for the origin of each participant's data (factor 1: health/descriptive social norm-based message condition vs. control condition, factor 2: healthy-weight vs. overweight, covariate: Study 1 or Study 2, dependent variable: fruit and vegetable intake).

For the health messages analysis, there was a significant main effect of condition [F (1, 171) = 6.62, *p* = .01, np^2^ = .04], no significant main effect of weight status [F (1, 171) = .72, *p* = .40, np^2^ = .004], no significant interaction between weight status and condition [F (1, 171) = 1.35, *p* = .25, np^2^ = .008], and no significant effect of study [F (1, 171) = .01, *p* = .92, np^2^ *≤* .001] in the ANOVA. Participants in the health message condition consumed 73.39 (SD = 45.80) grams of fruit and vegetables, compared to 51.91 (SD = 36.72) grams in the control condition (d = .52). See Table 5.

For the descriptive social norm-based messages analysis, there was a significant main effect of condition [F (1, 172) = 5.64, *p* = .02, np^2^ = .03], a significant main effect of weight status [F (1, 174) = 9.64, *p* = .002, np^2^ = .05], no significant interaction between weight status and condition [F (1, 172) = 1.03, *p* = .31, np^2^ = .006] and no significant effect of study [F (1, 172) = .07, *p* = .79, np^2^ *≤* .001] in the ANOVA. Participants in the descriptive social norm-based message condition consumed 66.07 (SD = 47.38) grams of fruit and vegetables, compared to 51.91 (SD = 36.72) grams in the control condition (d = .33). The main effect of weight status was explained by healthy-weight participants consuming 52.75 (SD = 38.65) grams of fruit and vegetables in comparison to 75.26 (SD = 49.11) grams by overweight participants. See Table 5.

The results of the meta-analysis indicate that when data were pooled across both studies, there was evidence that both health messages and descriptive social norm-based messages increased fruit and vegetable intake in comparison to control condition messages. The significant effects observed in our meta-analysis (but not consistently observed in individual study analyses) may be best explained by increased statistical power. The meta-analysis did not indicate that the effect of either message type interacted with participant weight status. This, alongside the small number of overweight children in Study 2, suggests that the interaction we observed between child weight status and message condition in Study 2 should be interpreted with caution.

## 5 General discussion

Across two studies we examined the effects of descriptive social norm-based messages about the fruit and vegetable consumption of other children and health messages on children's fruit and vegetable intake in comparison to control messages. Although we observed inconsistent findings across the two individual studies, when the data from studies 1 and 2 were pooled we found evidence that descriptive social norm-based messages had a small effect on fruit and vegetable intake, with children exposed to these messages eating more than children exposed to control messages. Likewise, we found that messages about the health benefits of eating fruit and vegetables significantly increased children's fruit and vegetable intake relative to control messages. Results from one of our studies (Study 2) were suggestive that health messages may only increase fruit and vegetable intake among healthy-weight children, but this result was not consistent across both studies or in the overall pooled analysis.

A number of studies in adults have found that exposure to descriptive social norm-based messages significantly increases fruit and vegetable consumption (Robinson et al., 2013, Robinson et al., 2013, Stok et al., 2014). However, in the current studies descriptive social norm-based messages produced relatively small changes to fruit and vegetable intake. A possible explanation for these results is that children may respond more strongly to context-specific eating norms. Context is likely to be an important factor which influences whether social norms influence behaviour. Studies investigating the influence of eating norms often expose participants to information about the eating behaviour of other people in the same context or setting (Burger et al., 2010, Pliner and Mann, 2004, Robinson et al., 2014, Robinson et al., 2014, Robinson et al., 2014, Robinson et al., 2014, Robinson, 2015). For example, Burger et al. (2010) exposed participants to the food choice of previous participants, and found that participants chose a snack consistent with what the previous participants had chosen. However, research in social psychology suggests that as normative information becomes less specific to a given context, the influence of that normative information on behaviour may decrease (Goldstein, Cialdini, & Griskevicius, 2008). In the current studies we exposed children to descriptive social norm information which was not directly relevant to the context children found themselves in, whereas, in previous work in which children have been socially influenced, there has been a shared context between influencers and children being influenced (Bevelander et al., 2012, Sharps and Robinson, 2015). Therefore, it is possible that children may find it more difficult to apply generalised normative beliefs (i.e. those devoid of context) about the behaviour of others to inform their food intake. To specifically address this, future studies could investigate whether context specific descriptive social norm-based messages regarding the fruit and vegetable intake of other children provide a stronger influence on eating behaviour than messages which are not context specific. There are, of course, other potential explanations for why the descriptive social norm-based messages in the present studies appeared to have only a small effect on eating behaviour (e.g. differences in study designs between the present studies and studies in adult populations), so further work will now need to specifically test whether children are more or less responsive to descriptive social norm-based messages than adults.

One factor which has shown to influence adherence to normative information is identification with the norm group. For example, Cruwys et al. (2012) showed that university students modelled the behaviour of an in group member (a student at the same university) but did not model the behaviour of an out group member (a student from another university). In the present studies, since descriptive social norm-based messages had not been investigated in children prior to this study, in Study 1 we examined messages regarding a general group i.e. ‘other children’. In Study 2 we altered the messages so that they referred to a group of a closer social proximity i.e. ‘children like you’. We did not measure how similar the children in the study felt to the children in the messages and this is a limitation of the present studies. It may be that a social reference group which is of a closer social proximity, or encouraging children to think about how they are similar to a social reference group, would provide a stronger influence of social norm-based messages on children's fruit and vegetable intake.

Previous studies have shown that weight status may affect the extent to which children copy the eating behaviour of their peers (Bevelander et al., 2012, Romero et al., 2009). It should be noted that a limitation of the present work was that we had a relatively small number of overweight participants in each of our studies, as well as when studies were combined in the meta-analysis, making it difficult to make firm conclusions about how weight status may affect how children respond to messages about fruit and vegetables. Given that childhood obesity has increased in recent times (Wang, McPherson, Marsh, Gortmaker, & Brown, 2011), future studies may benefit from understanding further differences in how healthy-weight and overweight children respond to healthy eating messages.

To date, the evidence regarding the effectiveness of health messages on promoting healthy eating is mixed (Bannon and Schwartz, 2006, Lapierre et al., 2011, Lawatsch, 1990, Maimaran and Fishbach, 2014, Robinson et al., 2013, Robinson et al., 2013). Our findings are consistent with research which showed a positive effect of health messages on intake (Bannon and Schwartz, 2006, Lawatsch, 1990, Robinson et al., 2013). Our findings build upon this research through showing the effectiveness of health messages in school-aged children (aged 6–11), whereas previous studies showed the effectiveness of health messages on food intake in pre-school children (Bannon and Schwartz, 2006, Lawatsch, 1990) and adults (Robinson, Harris, et al., 2013). However, our findings are in contrast to other research which has shown that health messages which present food as healthy, reduced intake (Maimaran & Fishbach, 2014). Maimaran and Fishbach (2014) presented crackers as healthy, and found that exposure to this message reduced children's selection of that food. In the current studies we presented fruit and vegetables as ‘healthy’, and found that these messages increased consumption of fruit and vegetables. A possible explanation for this difference may relate to the type of food which was presented as healthy. Research has shown that children have a good representation of the nutritional quality of food from a young age (Murphy, Youatt, Hoerr, Sawyer, & Andrews, 1995), therefore, it is plausible that health messages which reinforce the positive qualities of already assumed ‘healthy’ food, provide a benefit to consumption, whereas labelling a less nutritionally clear food as being healthy may compromise expected enjoyment or taste (Maimaran and Fishbach, 2014, Raghunathan et al., 2006).

In the present studies we examined the effectiveness of descriptive social norm-based messages, yet another type of norm which may be useful in behaviour change is an injunctive norm. Injunctive norms suggest the approval of others (Cialdini, Reno, & Kallgren, 1990). Research has shown that injunctive norms influence behaviour, and behavioural intentions (Cheng et al., 2008, Cialdini et al., 1990, Stok et al., 2014, Van Den Putte et al., 2005, Zaleski and Aloise-Younga, 2013). For example, in recent studies a lack of perceived parental emphasis on breakfast consumption was associated with breakfast skipping in adolescents (Cheng, Tereza et al., 2008), and injunctive norms were found to have a larger effect on intentions to stop smoking than descriptive norms (Van Den Putte et al., 2005). However, in another study no association was found between injunctive norms and fruit and vegetable, high calorie snack food, or sugar-sweetened beverage intake in adolescents (Lally et al., 2011). The majority of research investigating injunctive norms has been cross-sectional, or relied on self-reported intake, with no studies, to our knowledge, investigating the effectiveness of injunctive norms on children's eating behaviour in an experimental design. Further research examining whether other types of social norm-based information can motivate healthier eating in children would therefore be of value.

To our knowledge, these are the first studies to investigate whether descriptive social norm-based messages influence fruit and vegetable intake in children. However, the studies are not without limitations. Although we recruited relatively large samples in each study (n = 40 or more per experimental condition) and based our sample size calculation on a recent comparable study (Sharps & Robinson, 2015) both studies were underpowered to detect statistically small effect sizes. Based on the present findings and a recent meta-analysis of the size of effect that descriptive norms have on eating behaviour in adults (Robinson et al., 2014, Robinson et al., 2014), future studies examining the influence of descriptive norm messages in children or adults are likely to require larger sample sizes than has been common in this area of research. A further limitation was that the studies were conducted in a single experimental session with food intake measured immediately after message exposure, therefore, it is not possible to determine whether the effect of messages would be sustained over a longer period of time. The relatively small number of overweight children in both studies is also a limitation of the present research. We also examined consumption of two types of common fruit and vegetables which we presumed 6–11 year old UK children would be happy to consume, if they felt motivated to; carrots and grapes. It may be the case that descriptive social norm-based messages or health messages would act differently on the consumption of other (less liked) types of fruits and vegetables. Although our focus was on a specific age range of children (6–11 year olds) that have been shown to be responsive to social influence on eating behaviour in other studies (Sharps & Robinson, 2015), it may be the case that descriptive social norm-based messages about healthy eating would be more effective in older age ranges, as suggested by Stok et al., 2012, Stok et al., 2014. Furthermore, while we examined children's general liking of fruit and vegetables, we did not examine their liking of the specific test foods, which may have influenced their intake of the food. However, test food liking was not found to interact with exposure to social norm information in a previous study examining children's vegetable intake (Sharps & Robinson, 2015).

## 6 Conclusions

In conclusion, we conducted two experimental studies and found evidence that both health messages and descriptive social norm-based messages increased children's fruit and vegetable consumption relative to control messages. Whether descriptive social norm-based messages can be used to promote meaningful changes to children's dietary behaviour now warrants attention.

## Conflict of interest

The authors report no conflicts of interest.

## Figures and Tables

**Table 1:** Mean values (SDs) and statistical test results for BMI, age, gender, and beliefs about descriptive social norms for Study 1.

Variables	Descriptive social norm-based message (n = 49)	Health message (n = 48)	Control (n = 46)	Statistical test results
BMI (*z*-score)	.55 (1.23)	.99 (1.28)	.72 (1.31)	F(2, 140) = 1.46, *p* = .24, np^2^ = .02
Age (years)	8.67 (.97)	8.70 (.87)	8.88 (1.25)	F(2, 140) = .61, *p* = .55, np^2^ = .01
Gender (n)				
Males	18	21	18	X^2^ (2, n = 143) = .51, *p* = .77, r = .06.
Females	31	27	28
Beliefs about the fruit and vegetable intake of other children^a^	3.91 (.91)	3.50 (1.19)	3.87 (1.05)	F(2, 140) = 1.97, *p* = .14, np^2^ = .03

a A higher mean indicates that children believe that other children eat a large amount of fruit and vegetables on a scale of 1−5.

**Table 2:** Mean (SDs) fruit and vegetable intake and high calorie snack food intake for Study 1.

Condition	Fruit and vegetable intake (grams)	High calorie snack food intake (grams)
Descriptive social norm-based message (n = 49)	65.47 (40.77)	24.94 (20.51)
Health message (n = 48)	75.69 (47.13)^a^	25.50 (16.59)
Control (n = 46)	50.76 (29.54)^a^	25.26 (17.98)

a Indicates a significant difference at *p* < .01.

**Table 3:** Mean values (SDs) and statistical test results for BMI, age, gender, and beliefs about descriptive social norms for Study 2.

Variables	Descriptive social norm-based message (n = 41)	Health message (n = 41)	Exposure (n = 41)	Control (n = 41)	Statistical test results
BMI (*z*-score)	.76 (1.19)	.54 (1.09)	.40 (1.15)	.35 (.98)	F(3, 160) = 1.14, *p* = .33, np^2^ = .02
Age (years)	9.08 (1.25)	9.03 (1.22)	8.61 (1.39)	8.82 (1.35)	F(3, 160) = 1.12, *p* = .34, np^2^ = .02
Gender (n)					
Males	23	21	21	19	X^2^ (3, n = 164) = .78, *p* = .85, r = .07.
Females	18	20	20	22
Beliefs about the fruit and vegetable intake of other children^a^	4.46 (.71)	3.78 (.85)^b^	4.15 (.91)	3.71 (1.03)^b^	F(3, 160) = 6.44, *p* *≤* .001, np^2^ = .11

a A higher mean indicates that children believe that other children eat a large amount of fruit and vegetables on a scale of 1−5.

b Indicates a significant difference at *p <* .01 to the descriptive social norm-based message condition.

**Table 4:** Mean (SDs) fruit and vegetable intake and high calorie snack food intake for Study 2.

Condition	Fruit and vegetables	High calorie snack food
Descriptive social norm-based message (n = 41)	66.78 (54.76)	25.71 (17.84)
Health message (n = 41)	70.71 (44.62)	26.51 (17.63)
Exposure condition (n = 41)	66.78 (37.18)	22.66 (14.47)
Control (n = 41)	53.20 (43.77)	27.10 (18.82)

**Table 5:** Mean (SDs) fruit and vegetable intake for pooled data from studies 1 and 2.

Variables		Fruit and vegetable intake
Condition	Descriptive social norm-based message (n = 90)	66.07 (47.38)^a^
Health message (n = 89)	73.39 (45.80)^a^
Control (n = 87)	51.91 (36.72)
Weight-status (Descriptive social norm-based message condition only)	Healthy-weight children (n = 127)	52.75 (38.65)
Overweight children (n = 50)	75.26 (49.11)

a Indicates a significant difference at *p* < .05 to the Control condition.

## Footnotes

1 All children were able to correctly categorise the fruit and vegetable pictures.

2 We based this selection on how much children said they tended to eat in Study 1 and during the study no children disagreed or questioned the placement of the counter.
