# Supplementary material for: Encouraging children to eat more fruit and vegetables: Health vs. descriptive social norm-based messages
Source: Appetite. 2016 May 1;100:18–25. doi: 10.1016/j.appet.2016.01.031 (PMC4819560; doi:10.1016/j.appet.2016.01.031)
Supplement: Supplementary file 1 [file mmc1.docx]

Supplemental material

In order to examine whether weight-status, usual fruit and vegetable intake, and fruit and vegetable liking affected the effect of condition on the dependent variables, these variables were included as covariates in an ANCOVA. Furthermore, in order to examine whether child age, gender, fruit and vegetable liking, or usual fruit and vegetable consumption moderated any effects of message type on fruit and vegetable or high calorie snack food intake, we conducted a series of further analyses. We found no evidence that any of these variables moderated the effect of condition on fruit and vegetable, or high calorie snack food intake in any of the studies.

*Study 1*

One way ANCOVAs were conducted to examine the effect of condition on fruit and vegetable intake and high calorie snack food, when controlling for weight-status, usual fruit and vegetable intake, and fruit and vegetable liking in separate analyses. There was a significant main effect of condition on fruit and vegetable intake after controlling for weight-status, [F(2, 139)=4.27, *p*=.02, ƞp^2^ =.06], usual fruit and vegetable intake [F(2, 139)=4.56, *p*=.01, ƞp^2^ =.06], and fruit and vegetable liking [F(2, 139)=4.47, *p*=.01, ƞp^2^ =.06].

There was a no significant main effect of condition on high calorie snack food when controlling for weight-status [F(2, 139) = .002, *p* =.99, ƞp^2^ =<.001], usual fruit and vegetable intake [F(2, 139)=.01, *p*=.99, ƞp^2^ =<.001], and fruit and vegetable liking [F(2, 139)=.007, *p*=.99, ƞp^2^ =<.001]. Therefore, controlling for these variables did not alter the effect of condition on fruit and vegetable or high calorie snack food intake.

*Weight-status*

Two 3 (conditions) x 2 (weight-status; healthy-weight, overweight) ANOVAs were conducted. There was no significant main effect of weight-status on fruit and vegetable intake, [F(1, 137)=1.19, *p*=.28, ƞp^2^ =.009], or on high calorie snack food intake [F(1, 137)=.39, *p*=.53, ƞp^2^ =.003]. There was no significant interaction between condition and weight-status on fruit and vegetable intake [F(2, 137)=.17, *p*=.84, ƞp^2^ =.003], or high calorie snack food intake [F(2, 137)=.08, *p*=.92, ƞp^2^ =.001].

*Age*

Two 3 (condition) x 2 (age; younger (6-8 years), older (9-11 years) ANOVAs were conducted. There was no significant main effect of age on fruit and vegetable intake [F(1,137)=.89, *p*=.35, ƞp^2^ =.006], or on high calorie snack food intake [F(1,137)=1.45, *p*=.23, ƞp^2^ =.01]. There was no significant interaction between condition and age on fruit and vegetable intake [F(2,137)=.43, *p*=.65, ƞp^2^ =.006] or high calorie snack food intake [F(2,137)=1.68, *p*=.19, ƞp^2^ =.02].

*Gender*

Two 3 (condition) x 2 (gender) ANOVAs were conducted. There was no significant main effect of gender on fruit and vegetable intake [F(1,137)=.09, *p*=.76, ƞp^2^ =.001] or high calorie snack food intake [F(1,137)=1.47, *p*=.23, ƞp^2^ =.01]. There was no significant interaction between condition and gender on fruit and vegetable intake, [F(2, 137)=.01, *p*=.99, ƞp^2^ < .001], or high calorie snack food intake [F(2,137)=.47, *p*=.63, ƞp^2^ =.01].

*Fruit and vegetable liking*

Two 3 (condition) x 2 (fruit and vegetable liking; like (4 or 5 on the Likert scale) vs. dislike (1-3 on the Likert scale) ANOVAs were conducted. There was a significant main effect of fruit and vegetable liking on fruit and vegetable intake [F(1,137)=4.10, *p*=.05, ƞp^2^ =.03], whereby, children who liked fruit and vegetables ate more fruit and vegetables than children who did not like fruit and vegetables, t(141)=2.31, *p*=.02, *d*=80. There was no significant main effect of fruit and vegetable liking on high calorie snack food intake [F(2,137)=.002, *p*=.96, ƞp^2^ <.001]. In addition, there was no significant interaction between condition and fruit and vegetable liking on fruit and vegetable intake, [F(2,137)=1.33, *p*=.27, ƞp^2^ = .02], or high calorie snack food intake [F(2,137)=1.95, *p*=.15, ƞp^2^ =.03].

*Usual fruit and vegetable consumption*

Two 3 (condition) x 2 (usual fruit and vegetable intake; high (2 or more pieces of fruit and vegetables per day) vs. low (1 or no pieces of fruit and vegetables per day) ANOVAs were conducted. There was a significant main effect of usual fruit and vegetable intake on fruit and vegetable intake [F(1,137)=4.30, *p*=.04, ƞp^2^ =.03], whereby, usually high consumers of fruit and vegetables ate more fruit and vegetables than usually low consumers of fruit and vegetables, t(141)=2.11, *p*=.04, *d*=.35. There was no significant main effect of usual fruit and vegetable intake on high calorie snack food intake [F(1,137)=3.42, *p*=.07, ƞp^2^ =.02]. In addition, there was no significant interaction between condition and usual fruit and vegetable intake on fruit and vegetable intake, [F(2,137)=.60, *p*=.55, ƞp^2^ = .009], or on high calorie snack food intake [F(2,137)=.56, *p*=.58, ƞp^2^ =.008].

Study 2

One way ANCOVAs were conducted to examine the effect of condition on fruit and vegetable intake and high calorie snack food intake, when controlling for weight-status, usual fruit and vegetable intake, and fruit and vegetable liking. There was no significant main effect of condition on fruit and vegetable intake when controlling for weight-status [F(3, 159)=1.05, *p*=.37, ƞp^2^ =.02], usual fruit and vegetable intake [F(3, 159)=1.24, *p*=.30, ƞp^2^ =.02], or fruit and vegetable liking [F(3, 159)=1.25, *p*=.29, ƞp^2^ =.02].

There was no significant main effect of condition on high calorie snack food intake when controlling for weight-status [F(3, 159)=.53, *p*=.66, ƞp^2^ =.01], usual fruit and vegetable intake [F(3, 159)=.59, *p*=.62, ƞp^2^ =.01], or fruit and vegetable liking [F(3, 159)=.49, *p*=.69, ƞp^2^ .01]. Therefore, controlling for these variables did not alter the effect of condition on either fruit and vegetable or high calorie snack food intake.

*Age*

Two 4 (condition) x 2 (age; younger (6-8 years), older (9-11 years) ANOVAs were conducted. There was no significant main effect of age on fruit and vegetable intake [F(1, 156)=.07, *p*=.80, ƞp^2^ =<.001]. However, there was a significant main effect of age on high calorie snack food intake [F(1, 156)=4.83, *p*=.03, ƞp^2^ =.03], whereby, younger children ate more high calorie snack foods than older children, t(162)=1.97, *p*=.05, *d*=.32. There was no significant interaction between condition, and age on fruit and vegetable [F(3,156)=1.11, *p*=.35, ƞp^2^ =.02] or high calorie snack food intake [F(3,156)=1.37, *p*=.25, ƞp^2^ =.03].

*Gender*

Two 4 (condition) x 2 (gender) ANOVAs were conducted. There was no significant main effect of gender on fruit and vegetable intake [F(1,156)=.002, *p*=.97, ƞp^2^ <.001]. However, there was a significant main effect of gender on high calorie snack food intake [F(1,156)=7.28, *p*=.008, ƞp^2^ =.05], whereby, males ate more high calorie snack food than females, t(162)=2.68, *p*=.008, *d*=.42. There was no significant interaction between condition and gender on fruit and vegetable intake, [F(3, 156)=.92, *p*=.43, ƞp^2^ = .02], or high calorie snack food intake [F(3,156)=.72, *p*=.54, ƞp^2^ =.014].

*Fruit and vegetable liking*

Two 4 (condition) x 2 (fruit and vegetable liking; like (4 or 5 on the Likert scale), vs. dislike (1-3 on the Likert scale) ANOVAs were conducted. There was no significant main effect of fruit and vegetable liking on fruit and vegetable intake [F(1,156)=.23, *p*=.63, ƞp^2^ =.001] or high calorie snack food intake [F(1,156)=.36, *p*=.55, ƞp^2^=.002]. There was no significant interaction between condition and fruit and vegetable liking on fruit and vegetable intake, [F(3,156)=1.53, *p*=.21, ƞp^2^ = .03], or high calorie snack food intake [F(3,156)=.74, *p*=.53, ƞp^2^ =.014].

*Usual fruit and vegetable intake*

Two 4 (condition) x 2 (usual fruit and vegetable intake; high (2 or more pieces of fruit and vegetables per day), low (1 or no pieces of fruit and vegetables per day) ANOVAs were conducted. There was no significant main effect of usual fruit and vegetable intake on fruit and vegetable intake [F(1,156)=2.15, *p*=.15, ƞp^2^ =.01] or high calorie snack food intake [F(1,156)=.16, *p*=.69, ƞp^2^=.001]. There was no significant interaction between condition and usual fruit and vegetable intake on fruit and vegetable intake, [F(3,156)=.29, p=.83, ƞp^2^ = .006], or high calorie snack foods intake [F(3,156)=.32, *p*=.81, ƞp^2^ =.006].

**Table 1** Mean (SDs) for fruit and vegetable intake and high calorie snack food intake in healthy-weight and overweight children for study 2

| Condition | Healthy-weight  (n=127) | | Overweight  (n=37) | |
| --- | --- | --- | --- | --- |
|  | Fruit and vegetables | High calorie snack food | Fruit and vegetables | High calorie snack food |
| Descriptive social norm-based message (n=41) | 51.56 (42.58) | 22.70 (19.31) | 96.14 (64.75) | 31.50 (13.36) |
| Health message (n=41) | 74.12 (41.67)* | 25.53 (16.34) | 54.14 (57.73) | 31.29 (22.76) |
| Exposure condition (n=41) | 70.06 (39.82) | 25.00 (14.13) | 53.25 (19.75) | 13.00 (12.31) |
| Control (n=41) | 47.42 (39.14)* | 26.10 (18.37) | 77.00 (55.97) | 31.25 (21.35) |
